# Survey of the vaia storm deposits in the tegnas catchment (Dolomites, Italy): Field data and evidence of sediment-water flow types

**DOI:** 10.1016/j.dib.2020.106415

**Published:** 2020-10-18

**Authors:** Andrea Brenna, Nicola Surian, Massimiliano Ghinassi, Lorenzo Marchi

**Affiliations:** a Department of Geosciences, University of Padova, Padova, Italy; b Research Institute for Geo-hydrological Protection, National Research Council (CNR IRPI), Padova, Italy

**Keywords:** Flood deposits, Mountain streams, Debris flood, Geomorphological survey, Sedimentological survey, Field survey worksheet

## Abstract

Brenna et al. [1] developed a survey protocol to collect evidence aimed at classifying flood deposits on the basis of the flow type that mobilized and deposited sediment. Such a survey protocol was adopted to characterize the flood deposits in a mountain catchment of the Dolomites (the Tegnas Torrent and its tributaries; drainage area of 51 km^2^) after a high-magnitude hydrological event that occurred in October 2018 (the so-called “Vaia Storm”). In this article, we present the field data collected at thirty-two survey sites considering the geomorphological and sedimentological characteristics of the analysed sedimentary products and their effects on the vegetation. Data on the characteristics of the flood deposits have enabled recognizing the transport mechanisms that occurred during the Vaia Storm along the stream network [1]. Future applications of the survey protocol adopted in this study could compare and integrate the collected data with those presented in detail in this article.

## Specifications Table

**Table:** 

Subject	Earth-Surface Processes
Specific subject area	Flood deposits in mountain streams
Type of data	Figure Photograph Field survey worksheet - Excel file
How data were acquired	Geomorphological and sedimentological field survey of flood deposits
Data format	Raw
Parameters for data collection	Field data were collected following a standardized field survey protocol developed by Brenna et al. [1].
Description of data collection	Geomorphological and sedimentological evidence and effects on vegetation of flood deposit were collected through a field survey worksheet that allowed to objectively categorizing the study morpho-sedimentary products.
Data source location	Stream: Tegnas Torrent and its tributaries Geographical area: San Lucano valley and Angheraz valley; Dolomites Town/Region: Taibon Agordino, Belluno Province, Veneto Region Country: Italy The detailed location of the survey sites adopted for data collection is reported in Figure 1.
Data accessibility	With the article. Raw data (geomorphological, sedimentological and “effects on vegetation” characteristics of flood deposits) collected during the fieldwork at each survey site are reported in the field survey worksheets included in the Supplementary Material.
Related research article	Brenna, A., Surian, N., Ghinassi, M., Marchi, L., Sediment–water flows in mountain streams: Recognition and classification based on field evidence. *Geomorphology*, 371. https://doi.org/10.1016/j.geomorph.2020.107413

## Value of the Data

• Data presented in this paper derive from the application of a field-survey protocol that was developed to collect data concerning the morpho-sedimentary characteristics of flood deposits in mountain streams.
• Researchers interested in sediment-water flows in steep channels could be interested in both the survey protocol adopted for collecting the field data and the dataset presented in this article regarding different types of flood deposits occurring in a mountain stream and its steep tributaries.
• Data collected from future applications of the proposed survey protocol could be compared and integrated with those presented in detail in this article. A broad dataset of the characteristics of different flow-type deposits, possibly collected in different geological and geomorphological settings in response to different hydrological events, is crucial to improve our capability in recognizing different transport processes from post-flood analysis, with particular regard to sedimentary products of debris floods and hyperconcentrated flows.

## 1 Data Description

**Fig. 1:**
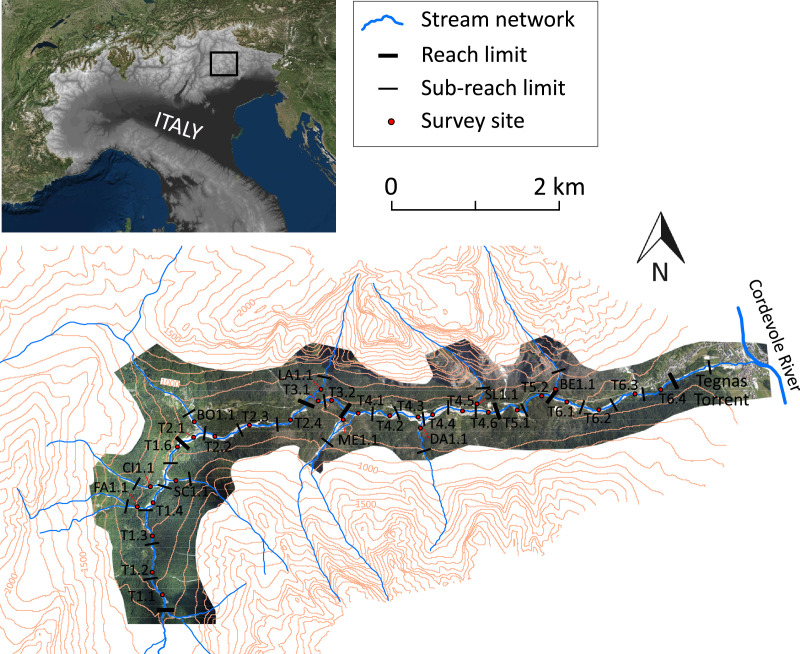
Location map and physiographic characteristics of the stream network. In the figure are reported the locations of the survey sites considered for the flood deposit description and field data collection. The bottom panel is modified from [1].

The data reported in this article derive from a detailed field survey of the flood deposits mobilized by the high-magnitude Vaia Storm (October 2018) along the main stem of the Tegnas Torrent and its tributaries (Dolomites, Italy) (Fig. 1). Details about the meteorological and hydrological features of the Vaia event, and the physiographic characteristics of the stream network are reported in [1]. Based on the survey protocol developed by Brenna et al. [1], we collected a series of geomorphological, sedimentological and “effects on vegetation” evidence characterizing each deposit that was surveyed during the fieldwork. Such evidence was employed to classify the sedimentary products on the basis of the sediment-water flow type (i.e., debris flow, hypeconcentrated flow, debris flood, water flow; see [1], and [2]) that transported and deposited a sedimentary body [1].

Thirty-two survey sites were considered in this study (i.e., 23 along the Tegnas Torrent, and 9 along the tributaries; see Fig. 1). A field survey worksheet was compiled for each site to describe the flood deposits, collecting data about the morphological configuration and landforms of the deposits, their sedimentological characteristics, and sedimentary structures, and the effects of sediment-water flows on the vegetation located within the channels and overbanks. See the following section (Experimental Design, Materials and Methods) for further details about the data collection.

All the compiled field survey worksheets are included in the Supplementary Material. For each survey site, we highlighted in yellow in the worksheet the field evidence arising from the description of a deposit. Among the 75 pieces of evidence considered in the worksheets, we recognized a broad spectrum of features during the fieldwork. At each survey site was identified a number of evidence ranging from 16 to 22. Two exceptions are represented by sites T3.1 and T3.2, where the lack of fully developed depositional bodies permitted to recognize only 4 and 5 pieces of evidence, respectively. The distribution of the evidence recognized at each survey site was used to classify the deposits on the basis of the flow types [1]. In each field survey worksheet we highlighted the flow type identified by Brenna et al. [1] as responsible for the sedimentation of deposits analyzed.

## 2 Experimental Design, Materials and Methods

**Fig. 2:**
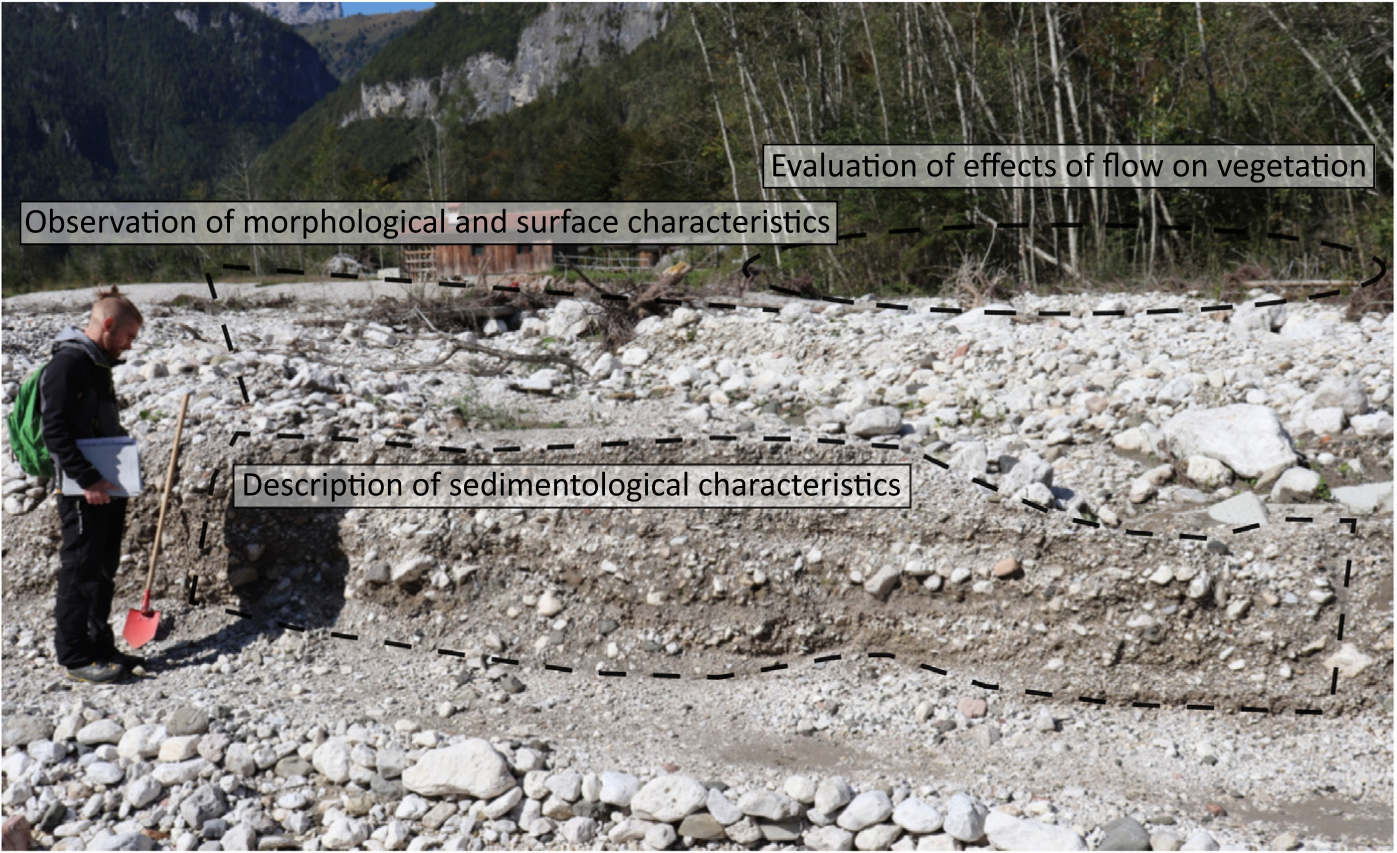
Example of a survey site considered during the fieldwork for description of the flood deposits.

The detailed field survey of the Vaia event deposits was conducted between May and August 2019 along the 12.9 km stretch of the stream network. The Tegnas main stem and the tributaries were segmented following procedures available in literature ([3, 4], and [5]) obtaining homogeneous sub-reaches with lengths of about 200–500 m (Fig. 1). For each sub-reach, we identified one survey site where the surface and sub-surface features of the deposits mobilized by the Vaia event could be characterized according to the protocol proposed by Brenna et al. [1], gathering the information included in the field survey worksheet (Fig. 2). The exposed sections considered for the sedimentological description of the deposits had an area ranging from 2 to 10 m^2^. The 75 geomorphological characteristics, effects on vegetation, and sedimentological characteristics considered during the survey, which are pieces of evidence indicating different flow types, are reported in the field survey worksheets (Supplementary Material) and in [1].

## Declaration of Competing Interest

The authors declare that they have no known competing financial interests or personal relationships which have, or could be perceived to have, influenced the work reported in this article.

## Appendix Supplementary materials

Image, application 1

## Footnotes

Supplementary material associated with this article can be found in the online version at doi:10.1016/j.dib.2020.106415.
